# Evaluation of the Composite Mechanism of Nano-Fe_2_O_3_/Asphalt Based on Molecular Simulation and Experiments

**DOI:** 10.3390/ma14123425

**Published:** 2021-06-21

**Authors:** Yuhao He, Qing Zeng, Yaru Liu, Peng Liu, Yuqin Zeng, Zhenghong Xu, Qicheng Liu

**Affiliations:** 1 School of Materials Science and Engineering, Changsha University of Science & Technology, Changsha 410114, China; heyuhao_csust@163.com (Y.H.); liupengmse@csust.edu.cn (P.L.); 13955427279@163.com (Z.X.); qichengliu1856@163.com (Q.L.); 2 School of Physics & Electronic Science, Changsha University of Science & Technology, Changsha 410114, China; ZengQing@stu.csust.edu.cn (Q.Z.); zengyq1996@hotmail.com (Y.Z.); 3 Hunan Provincial Key Laboratory of Flexible Electronic Materials Genome Engineering, Changsha 410114, China; 4 School of Traffic & Transportation Engineering, Changsha University of Science & Technology, Changsha 410114, China

**Keywords:** nano-Fe_2_O_3_, asphalt, AFM, molecular simulation, radial distribution function

## Abstract

Asphalt, as an indispensable binder in road paving, plays an important role in transportation development. However, the mechanism of action between the modifier and asphalt cannot be fully explained by the existing test methods. This paper combines molecular simulations with experiments to provide a research and analysis tool to evaluate the “structure−performance” relationship of asphalt. From the trend of experimental results, the optimal content of Nano-Fe_2_O_3_ is 1% to 3%. The AFM micrograph of the asphalt material shows that at 3%, the Nano-Fe_2_O_3_ can be effectively dispersed in the asphalt and the unique “ bee structures “ of the asphalt can be adsorbed around the modifier. Molecular dynamics studies and results show that when Nano-Fe_2_O_3_ are incorporated into the asphalt and have a strong adsorption force on the colloidal structure of asphalt, the “ bee structures “ can be adsorbed around the Nano-Fe_2_O_3_. In the range of 208–543 K, the sol-gel structure of asphalt in the Nano-Fe_2_O_3_/asphalt composite system is gradually disrupted.

## 1. Introduction

In recent years, more and more researchers are using nanomaterials to improve the drawbacks arising from the use of asphalt and asphalt composites [1].

Liao [2] et al. improved the resistance to aging under UV light by incorporating Nano-TiO_2_ into asphalt binders. Their results showed that the incorporation of Nano-TiO_2_ had a positive effect on the aging of asphalt binders, reducing the rate of aging as well as improving the asphalt road performance [3]. They added different ratios of TiO_2_ and ZnO to the asphalt binder and exposed the modified asphalt to sunlight for a long enough period of time. It has been shown that the temperature of TiO_2_ and ZnO modified asphalt was on average 1.88 °C lower than that of unmodified one [4]. In both samples containing 2% and 4% nanomaterials, the free energy of adhesion of the asphalt binder was increased, which made more energy required to separate the asphalt binder from the aggregate surface [5]. A. Ābele [6] modified RTFOT with recycled polymers to show better low-temperature performance, as well as greater resistance to rutting and fatigue. The experimental results showed that the application of nanomaterials reduced the debonding energy and was thermodynamically more stable. The experimental results with different nano-modifier dosing showed that the improvement of peel resistance of asphalt mixes was more significant with nano-Fe_2_O_3_.

Up to now, very few studies have explored the interaction between the modifier and asphalt. Peng Wang [7] chose the SARA component structure model to study the interactions of asphaltenes, saturated fractions, aromatic fractions, and gums. The results show that van der Waals is the main force controlling the intermolecular interactions. The arrangement of SARA fractions for intermolecular interactions in asphalt is essentially consistent with modern colloid theory. Albert M. Hung [8] showed by simulations and computational analysis that the interactions between wax and asphalt components (<22 kcal/mol) are much weaker than those of the asphalt components themselves (>50 kcal/mol). With the availability of molecular simulation techniques, it is possible to bring our study of asphalt composites to a smaller scale for cross-scale studies.

With the advent of modern chromatographic techniques, Corbett [9] proposed elution-adsorption liquid chromatography of activated alumina with solvents of increasing polarity and aromaticity separate components other than asphaltenes into saturates, aromatics, and gums. As a result of this method, the composition of bitumen is usually divided into saturates, aromatics, gums, and asphaltenes, the so-called SARA fraction [10].

However, the effect of nanoparticles on the macroscopic properties of asphalt composites and their modification mechanisms are still unclear. In this work, we started from the question of whether nano-Fe_2_O_3_ can enhance the performance of asphalt and the uncertain mechanism of interaction between nano-Fe_2_O_3_ and asphalt. Molecular simulation of nano-Fe_2_O_3_ with asphalt using molecular dynamics aims to reveal the effect of nano-Fe_2_O_3_ on asphalt properties as well as to explain the interaction mechanism between nano-Fe_2_O_3_ and asphalt at the molecular level. It overcomes the problem of asphalt as an extremely complex mixture, and the mechanism of interaction between nano-Fe_2_O_3_ and asphalt is difficult to be revealed by conventional detection means. It also bridges the gap of the current research in this area and promotes the better application of molecular simulation methods in the field of asphalt.

## 2. Experimental and Simulation Calculation Details

### 2.1. Experimental Details

**Figure 1:**
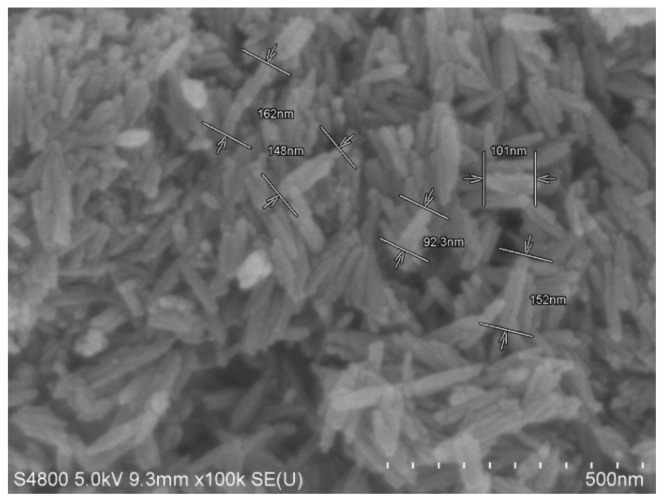
SEM image of Nano-Fe_2_O_3_.

The 70# matrix asphalt (Hunan Baoli Asphalt Co., Ltd, Changsha, China ) was placed in the oven at 130 °C for 1 h. After the matrix asphalt had certain fluidity, the matrix asphalt was placed in a beaker according to the amount required for the experiment. The beaker was placed in an oil bath and heated to 150 °C with constant stirring until the asphalt was in a molten homogenize state. Nanoscale α-Fe_2_O_3_ was used as the modifier. The Nano-Fe_2_O_3_ were added to the asphalt in different doses and homogenized with the matrix asphalt in a high homogenizer at a temperature of 150 °C at 6000 r/min for 45 min. The prepared Nano-Fe_2_O_3_/asphalt composites were poured into the molds required for testing and cooled to test conditions, and the rest of the composites were cooled to room temperature for subsequent testing. The nanoparticle characteristics are shown in the SEM image (Hitachi S-4800, Hitachi, Tokyo, Japan ) in Figure 1. The size is about 90–150 nm.

### 2.2. Simulation Calculation Details

Saturated hydrocarbons are shown in Fourier transform infrared spectroscopy (FTIR) to contain different branched structures and some long aliphatic chains and small amounts of cycloalkanes with traces of polar atoms and aromatic rings. It usually represents 5 to 15 wt.% of the total asphalt [9,11,12]. Asphalt contains more than 40% to 50% of saturated aromatic hydrocarbons, and they are mostly a mixture of hydrocarbons and non-compounds. The term mixture of hydrocarbons refers to the presence of cycloalkane rings and alkyl side chains and aromatic rings in the same molecule. Gums are also known as polar aromatic hydrocarbons, with a lower structure of compound aromatic hydrocarbons. Gums usually contain two to four fused rings and are sometimes more polar than asphaltenes [13,14,15]. The molecular structure of asphaltenes, compared to other components in asphalt, has been the focus of research on asphaltene materials in the last decade, leading to the gradual identification of representative molecular models for asphaltenes. Asphaltene H/C ratios range from 0.98 to 1.56 [16], and as shown by UV fluorescence [12,14], FTIR [12,14], X-ray Raman spectroscopy [17], and NMR [15,18,19], the molecular model of asphaltenes contains fused aromatic rings with a maximum value of the UV fluorescence spectrum around 450 nm [14], then the possible structures correspond to 4 to 10 aromatic rings and a certain amount of aliphatic chains. Many polar groups are also present in asphalt molecules [20], but the presence of molten aromatic structures is still a fundamental feature that distinguishes asphalt molecules from others [21]. In this paper, we use the Yen-Mullins [14,21,22] molecular model, which is characterized by the sulfur atom located in the thiophene ring and the construction of some aliphatic chains.

**Figure 2:**
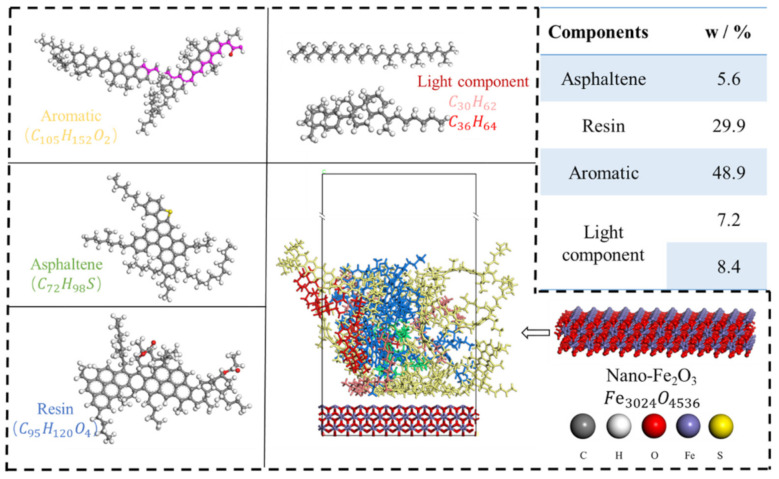
Structural model of asphalt SARA and Nano-Fe_2_O_3_.

In contrast, the Nano-Fe_2_O_3_ is intercepted with a certain thickness of the model to eliminate the error of uneven charge distribution caused by the thinness of the Nano-Fe_2_O_3_. The detailed molecular composition is shown in Figure 2.

In this paper, the models of asphalt and asphalt/iron oxide nanoparticles were selected for structural optimization, annealing, and radial distribution function (RDF) study based on molecular dynamics simulations with NPT tethered clone, COMPASS Ⅱ force field, and Ewald summation method as the computational conditions.

On the constructed asphalt model, the structure is first annealed, and the simulation calculation parameters are generally set to 15 annealing cycles at NPT, 298 K–500 K. After the annealing treatment, the cell length of the constructed asphalt colloidal system model was stabilized between 31.5 and 32 Å, and the density was stabilized between 0.90 and 0.94. The annealed optimized structure was then subjected to molecular dynamics simulations in NPT and NVE for 900 ps to allow the structure to reach a steady state.

## 3. Results and Discussion

### 3.1. Evaluation of Basic Physical Properties

From Table 1, it can be seen that the softening point of asphalt composites increases with the increasing amount of Nano-Fe_2_O_3_ doping, while the ductility and needle penetration are opposite to each other. It is also reflected from the basic physical properties that the incorporation of Nano-Fe_2_O_3_ can enhance the high-temperature stability of asphalt composites.

With the increasing amount of Nano-Fe_2_O_3_ doping, the increase of softening point before 1–3% is greater than that of 4–5%. The penetration decreases significantly with the increase of Nano-Fe_2_O_3_ doping, stabilizes at the content of 1–3%, and then continues to decrease with the increase of Nano-Fe_2_O_3_ content. The ductility gradually decreases with the increase of Nano-Fe_2_O_3_ doping and only decreases between 2% and 3% of the content. This phenomenon may occur because the incorporation of Nano-Fe_2_O_3_ gradually interacts with the stable asphalt colloidal structure. This results in a gradual increase in the softening point of the asphalt and a decrease in the penetration and ductility properties. Overall, the optimal content of Nano-Fe_2_O_3_ is 2% to 3%.

### 3.2. Viscosity

**Figure 3:**
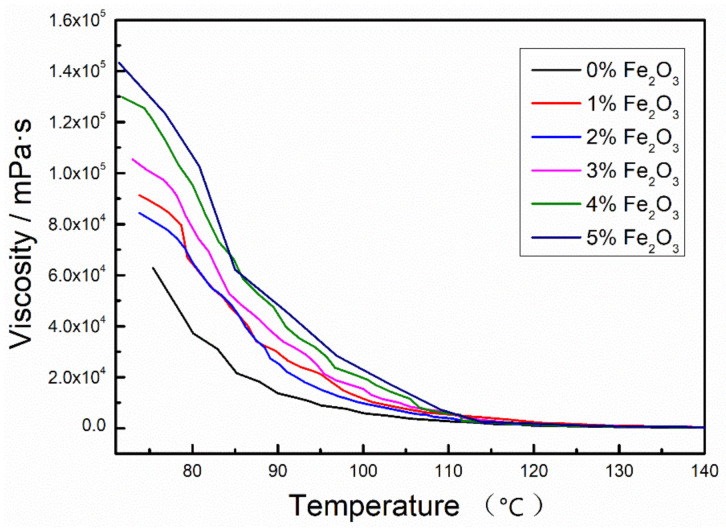
Effect of different Nano-Fe_2_O_3_ on asphalt viscosity at different temperatures.

Figure 3 shows the effect of different Nano-Fe_2_O_3_ doping levels on the viscosity of asphalt at different temperatures. From the figure, it can be seen that the viscosity of asphalt increases with the addition of modifier Nano-Fe_2_O_3_. The viscosity of Nano-Fe_2_O_3_/asphalt composites has a large increase compared to the matrix asphalt at the doping level of 1–3%. This is because the incorporation of Nano-Fe_2_O_3_ can effectively interact with the asphalt colloid, such as charge attraction, which leads to the difficulty of the components of Nano-Fe_2_O_3_/asphalt composites to move with the increase of temperature, which also increases the viscosity of the composites. With the increase of Nano-Fe_2_O_3_ doping amount up to 4–5%, the viscosity of asphalt large changed more significantly compared to 1–3%. This also indicates that when Nano-Fe_2_O_3_ reaches large dosing, the factors affecting the viscosity of asphalt are not only the interaction between the modifier and the asphalt colloid but also a large amount of Nano-Fe_2_O_3_ will exist within the asphalt system, leading to an increase in friction within the Nano-Fe_2_O_3_/asphalt composite system.

### 3.3. Activation Energy of Viscous Flow

The viscosity η of asphalt at different temperatures T is plotted against $\ln\left(\eta \right)$ and 1T. The slope of the resulting line is the value of the coefficient of 1T, which is the value of EaR, where R is the universal gas constant (8.314$\;J\cdot {\mathrm{mol}}^{-1}\cdot K^{-1}$), which in turn leads to the $E_{a}$ of the Nano-Fe_2_O_3_/asphalt composite.

As shown in Table 2, with the incorporation of Nano-Fe_2_O_3_, the $E_{a}$ values of asphalt composites showed a trend of decreasing before increasing. The smallest $E_{a}$ value was 6.96 $\mathrm{kJ}\cdot {\mathrm{mol}}^{-1}$ at 1% doping. With the increase of doping amount, the $E_{a}$ value stabilized at 2–3% doping amount. When Nano-Fe_2_O_3_ reaches high dosing (4–5%), the $E_{a}$ values increase again, to 7.93 $\mathrm{kJ}\cdot {\mathrm{mol}}^{-1}$, 7.78 $\mathrm{kJ}\cdot {\mathrm{mol}}^{-1}$, and even exceed the 7.16 $\mathrm{kJ}\cdot {\mathrm{mol}}^{-1}$ of the matrix asphalt. This shows that the matrix asphalt is more flowable and flexible at 1% of Nano-Fe_2_O_3_, and the asphalt components require less energy to move and have improved temperature sensitivity. The amount of admixture that does not differ much from this is 2% to 3%.

### 3.4. FTIR Analysis

In this paper, FTIR tests were performed on the Nano-Fe_2_O_3_/asphalt composite samples, as shown in Table 3, to resolve the main absorption peaks in the Nano-Fe_2_O_3_/asphalt composite.

**Figure 4:**
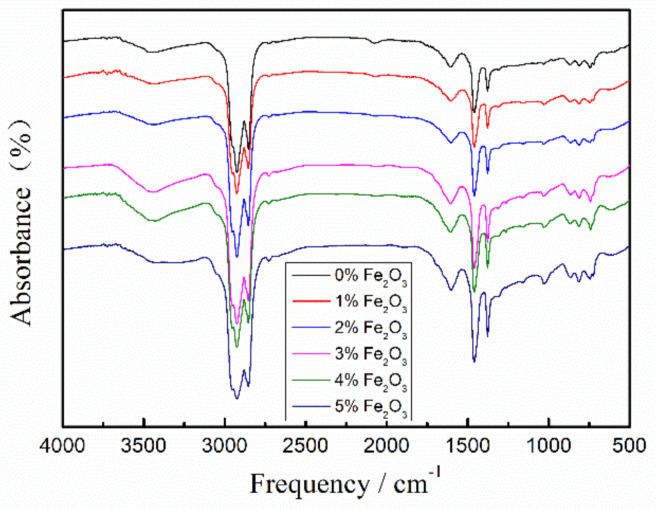
FTIR maps of modified asphalt with different Nano-Fe_2_O_3_ doping.

As shown in Figure 4, the characteristic peaks of asphalt at 1463 cm^−1^ and 1375 cm^−1^ are caused by the bending vibration of the -CH_2_ group in the light component. Additionally, 2920 cm^−1^ and 2852 cm^−1^ are the characteristic peaks of the stretching vibration of the -CH_2_ group in the light component. The characteristic peaks of stretching vibrations caused by C = C on the asphalt aromatic ring are at 1600 cm^−1^ and 1480 cm^−1^. Additionally, the characteristic peak at 710 cm^−1^ is generated by the bending vibration of the alkyl group.

With the doping of Nano-Fe_2_O_3_, a relatively weak characteristic peak appears at 1157 cm^−1^, which is due to the stretching vibration of the C-O bond. The characteristic peaks of the stretching vibration of alkyl sulfoxide (R_2_SO) and aryl sulfoxide (S=O) at 1030 cm^−1^, while the sulfoxide is mainly a dehydrogenation product the components in asphalt.

The incorporation of Nano-Fe_2_O_3_ did not shift the characteristic peaks of the asphalt, and only the intensity was changed. Together with the changes in the intensity of the characteristic peaks at 1157 cm^−1^ and 1030 cm^−1^. Therefore, it can be speculated that the interaction between Nano-Fe_2_O_3_ and the components of asphalt may be mainly physical.

### 3.5. Microstructure Analysis of AFM

**Figure 5:**
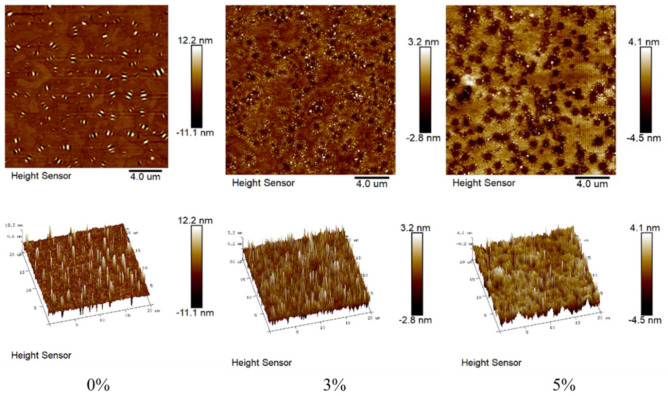
Microscopic characterization of AFM at different doping levels.

The two-dimensional microscopic morphology in Figure 5 shows that the matrix asphalt has clear “bee structures” with a clear boundary of the structural inclusion phase. This is because the “bee structures” are formed by the co-crystallization of microcrystalline waxes with asphaltenes as the core [7].

When the amount of Nano-Fe_2_O_3_ doping is 3%, the two-dimensional microstructure shows that the number of “bee structures” characteristic of asphalt decreases, and the “bee structures” become smaller, and most of them gradually. The number of “bee structures” decreases, the “bee structures” become smaller, and most of them are steadily clustered around Nano-Fe_2_O_3_. Similarly, it can be seen that the surface of asphalt becomes rough due to the incorporation of Nano-Fe_2_O_3_, but the peaks and valleys of “bee structures” do not disappear but become smaller peaks and valleys. Therefore, we can speculate that the addition of Nano-Fe_2_O_3_ destroyed the original “bee structures” of the asphalt and formed finer “bee structures”.

The diameter of Nano-Fe_2_O_3_ increases at 5% doping, probably due to the aggregation of Nano-Fe_2_O_3_ when it reaches a high doping level. This is probably due to the aggregation of Nano-Fe_2_O_3_ when it reaches a high doping level, and there are fewer “bee structures” around Nano-Fe_2_O_3_. From the three-dimensional microstructure, the roughness increased due to the aggregation of Nano-Fe_2_O_3_, compared to 3%.

This may be because the incorporation of Nano-Fe_2_O_3_ interacts with the asphalt colloid to reduce the number of microcrystalline waxes used to aggregate around the asphaltene and make it difficult for the asphalt colloid to agglomerate on a large scale. As a result, it is difficult to form obvious and large “bee structures” in the asphalt colloid system, but instead, similar structures like black and white spots are formed.

In terms of colloid theory, the more uniformly dispersed the colloid is, the better its macroscopic properties will be, which also confirms the excellent macroscopic properties of Nano-Fe_2_O_3_ modified asphalt.

### 3.6. RDF Analysis Based on Molecular Simulation

**Figure 6:**
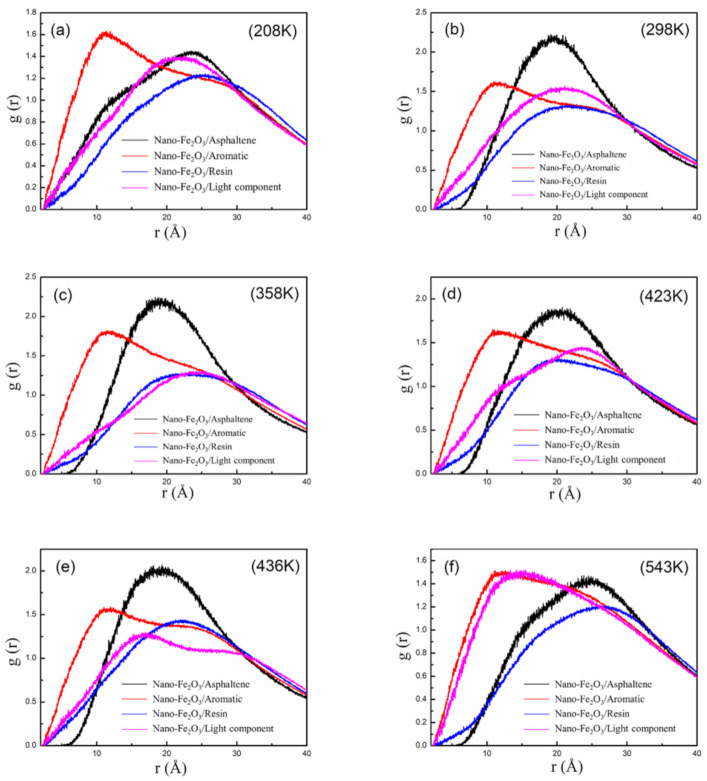
RDF between Nano-Fe_2_O_3_ and asphalt components in Nano-Fe_2_O_3_/asphalt composite system. Figures (**a**–**f**) are RDF curves at different temperatures.

As shown in Figure 6, when Nano-Fe_2_O_3_ was incorporated into the asphalt, the sol-gel structure of the asphalt within the Nano-Fe_2_O_3_/asphalt composite system was gradually disrupted at temperatures from 208 K to 543 K. With the increasing temperature, the asphaltene shrank from a distance of 23 Å to 19 Å from Nano-Fe_2_O_3_. When the temperature was raised to 543 K, the distance between the asphaltene and Nano-Fe_2_O_3_,this suggests that the addition of iron oxide causes the colloidal structure of the asphaltene to move toward the Nano-Fe_2_O_3_ as the temperature increases and forms the surface interface of the Nano-Fe_2_O_3_/asphalt composite system. In contrast, the resin keeps aggregating within 10 Å from Nano-Fe_2_O_3_.

As far as the colloidal structure of asphalt is concerned, the aromatic fraction and resin molecules are mainly clustered around the asphaltene. Most of these three components contain a benzene ring structure, thus creating a “π-π” stacking effect. Subsequently, they are distributed among the light components in this state. When Nano-Fe_2_O_3_ is incorporated into the asphalt as a modifier, with the increase of temperature, asphaltene, aromatic fraction, and resin gradually move to the surface of Nano-Fe_2_O_3_, forming the interaction between the surface charge of Nano-Fe_2_O_3_ and the aromatic ring. Additionally, the aromatic fractions gathered around the asphaltene change to gather around Nano-Fe_2_O_3_. Additionally, when the temperature reaches the high-temperature state (356–543 K), the vibration rate of each component molecule within the asphalt colloidal system is large, and the asphaltene also gains more molecular vibration kinetic energy because of the increase in temperature, and can even cross the energy barrier between the asphaltene and the molecules adsorbed around it, so that the colloidal structure of asphalt will be destroyed, and thus will gradually approach the surface of Nano-Fe_2_O_3_. This is also reflected in the AFM diagram; when the iron oxide is incorporated into the asphalt, the “bee structures” will gather around the Nano-Fe_2_O_3_, and the “bee structures” will become smaller, and the height will also the “bee structures” become smaller and their height decreases.

## 4. Conclusions

In the Nano-Fe_2_O_3_/asphalt composites prepared by the high-temperature melting method, the optimal amount of Nano-Fe_2_O_3_ is 1–3%. Nano-Fe_2_O_3_ can effectively improve the physical properties of asphalt.

From the AFM diagram, it can be seen that Nano-Fe_2_O_3_ can be effectively dispersed in the matrix asphalt. The interaction between the asphalt “bee structures” and Nano-Fe_2_O_3_ is especially obvious at 3% compared with 5%, and it is wrapped around. In this case, the aggregation of the asphalt is effectively prevented, and its molecular movement is hindered. Thus, the physical properties of asphalt composites can be improved. It can be speculated from FTIR that the interaction between Nano-Fe_2_O_3_ and each component of asphalt may be mainly physical.

When Nano-Fe_2_O_3_ was incorporated into asphalt, the solvation structure of asphalt within the Nano-Fe_2_O_3_/asphalt composite system was gradually destroyed at 208 K–543 K. The asphaltene shrank from the distance bee structures 23 Å to 19 Å. When the temperature was increased to 543 K, the distance between asphaltene and Nano-Fe_2_O_3_ increased to 25 with the increase of temperature, the addition of Nano-Fe_2_O_3_ causes the colloidal structure of asphalt to move toward Nano-Fe_2_O_3_ and forms the surface interface of the Nano-Fe_2_O_3_/asphalt composite system.

## Figures and Tables

**Table 1:** Effect of different content of Nano-Fe_2_O_3_ on the properties of asphalt composites.

Nano-Fe_2_O_3_ (%)	Softening Point (°C)	Penetration Degree (25 °C, 0.1 mm)	Ductility (5 °C, cm)
0	48.6	67.1	73
1	50.2	59.5	65
2	51.2	60.6	52
3	52	60.7	47
4	52.2	56.4	35
5	52.5	54.1	24

**Table 2:** Effect of different doping of Nano-Fe_2_O_3_ on E_a of asphalt composites.

Nano-Fe_2_O_3_%	lnη−1T	$E_{a}$ $/kJ\cdot mol^{-1}$
0	y=897.07x−0.18	7.46
1	y=837.02x+0.79	6.96
2	y=912.53x−0.17	7.59
3	y=894.93x+0.24	7.44
4	y=954.29x−0.33	7.93
5	y=936.23x−0.18	7.78

**Table 3:** Spectral analysis of main absorption peaks in asphalt composites.

Absorption Peaks	Functional Groups
2924 cm^−1^	Asymmetric stretching vibration of methylene, (C–H)
2853 cm^−1^	Symmetric stretching vibration of methylene, (C–H)
1601 cm^−1^	Respiratory vibration of asymmetrically substituted benzene rings
1460 cm^−1^	Shear vibration of methylene, (–CH_2_–)
1377 cm^−1^	Umbrella vibration of methyl, (–CH_3_)
1030 cm^−1^	Stretching vibration of sulfoxide group, (S=O)
868 cm^−1^	Stretching vibration of benzene ring
813 cm^−1^	Stretching vibration of benzene ring
745 cm^−1^	Bending vibrations of aromatic branched chains
724 cm^−1^	Co-vibration of methylene chain segment, (CH_2_)_n_ (*n* ≥ 4)

## Data Availability

The data presented in this study are available on request from the corresponding author.
